# A methodological primer of extracellular vesicles isolation and characterization via different techniques

**DOI:** 10.1093/biomethods/bpae009

**Published:** 2024-02-13

**Authors:** Farhang Aliakbari, Noah B Stocek, Maxximuss Cole-André, Janice Gomes, Giovanni Fanchini, Stephen H Pasternak, Gunna Christiansen, Dina Morshedi, Kathryn Volkening, Michael J Strong

**Affiliations:** Molecular Medicine Group, Robarts Research Institute, Schulich School of Medicine and Dentistry, University of Western Ontario, London, Ontario N6A 3K7, Canada; Department of Physics and Astronomy, University of Western Ontario, London, Ontario N6A 3K7, Canada; Department of Physics and Astronomy, University of Western Ontario, London, Ontario N6A 3K7, Canada; Molecular Medicine Group, Robarts Research Institute, Schulich School of Medicine and Dentistry, University of Western Ontario, London, Ontario N6A 3K7, Canada; Department of Physics and Astronomy, University of Western Ontario, London, Ontario N6A 3K7, Canada; Department of Chemistry, University of Western Ontario, London, Ontario N6A 3K7, Canada; Molecular Medicine Group, Robarts Research Institute, Schulich School of Medicine and Dentistry, University of Western Ontario, London, Ontario N6A 3K7, Canada; Department of Clinical Neurological Sciences, Schulich School of Medicine and Dentistry, University of Western Ontario, London, Ontario N6A 3K7, Canada; Department of Health Science and Technology, The Faculty of Medicine, Medical Microbiology and Immunology, Aalborg University, Aalborg Ø 9220, Denmark; Bioprocess Engineering Department, Institute of Industrial and Environmental Biotechnology, National Institute of Genetic Engineering and Biotechnology, Tehran, P.O. Box 14965/161, Iran; Molecular Medicine Group, Robarts Research Institute, Schulich School of Medicine and Dentistry, University of Western Ontario, London, Ontario N6A 3K7, Canada; Department of Clinical Neurological Sciences, Schulich School of Medicine and Dentistry, University of Western Ontario, London, Ontario N6A 3K7, Canada; Molecular Medicine Group, Robarts Research Institute, Schulich School of Medicine and Dentistry, University of Western Ontario, London, Ontario N6A 3K7, Canada; Department of Clinical Neurological Sciences, Schulich School of Medicine and Dentistry, University of Western Ontario, London, Ontario N6A 3K7, Canada

**Keywords:** exosome, extraction, PEG 8000, polymer, ultracentrifugation, ultrafiltration

## Abstract

We present four different protocols of varying complexity for the isolation of cell culture-derived extracellular vesicles (EVs)/exosome-enriched fractions with the objective of providing researchers with easily conducted methods that can be adapted for many different uses in various laboratory settings and locations. These protocols are primarily based on polymer precipitation, filtration and/or ultracentrifugation, as well as size-exclusion chromatography (SEC) and include: (i) polyethylene glycol and sodium chloride supplementation of the conditioned medium followed by low-speed centrifugation; (ii) ultracentrifugation of conditioned medium; (iii) filtration of conditioned media through a 100-kDa exclusion filter; and (iv) isolation using a standard commercial kit. These techniques can be followed by further purification by ultracentrifugation, sucrose density gradient centrifugation, or SEC if needed and the equipment is available. HEK293 and SH-SY5Y cell cultures were used to generate conditioned medium containing exosomes. This medium was then depleted of cells and debris, filtered through a 0.2-µM filter, and supplemented with protease and RNAse inhibitors prior to exosomal isolation. The purified EVs can be used immediately or stably stored at 4°C (up to a week for imaging or using intact EVS downstream) or at −80°C for extended periods and then used for biochemical study. Our aim is not to compare these methodologies but to present them with descriptors so that researchers can choose the “best method” for their work under their individual conditions.

## Introduction

Exosomes are a type of membrane-bound biological nanovesicle with a diameter of 20–150 nm that is generated primarily from the endocytic membrane and released by exocytosis. They were first identified in the early 1980s as small membrane vesicles released from reticulocytes that contained the transferrin receptor (TfR) [1–3] and have been shown to regulate biological processes in a paracrine or endocrine manner and to transport a variety of cargo [4, 5], including messenger RNA (mRNA), microRNA (miRNA), proteins, and lipids [6]. Because of this, exosomes have attracted attention in the field of therapy and diagnosis [7–11] including their use as nanocarriers to transport drug/cargo to the target site [12], therapeutic applications [13] and as biomarkers [8, 14].

Numerous disorders, including a broad range of cancers, neurodegenerative disorders and infectious diseases can be diagnosed with the help of the identification and characterization of exosomes [15–24]. Exosomes have been proposed to be involved in mediating both physiological and pathological functions [25] including the immune response [26], regeneration [11, 27, 28], blood clotting, stem cell preservation, and are also likely involved in cell-to-cell communication [29, 30]. In pathological conditions, exosomes have been implicated in several processes including tumorigenesis, tumor angiogenesis, metastasis, and the development of drug resistance [25].

Exosomes can be identified and characterized by several methods including the detection of specific protein markers [e.g. CD9, CD63, CD81, CD82, CD29, Hsp20, Hsp60, Hsp70, Hsp90, TSG101, Alix, Syntenin-1, caveolin-1, and MHCI and MHCII for antigen-presenting cells (APCs)], morphology [electron microscopy including transmission electron microscopy (TEM), scanning electron microscopy, and cryo-electron microscopy (cryo-EM), as well as atomic force microscopy (AFM)], and size characterization [Nanoparticle tracking analysis and dynamic light scattering (DLS)] [25]. Moreover, exosomes can also be labeled by fluorescent dyes and probes which can be used to track their uptake into cells (e.g. Mem Dye-Green, Mem Dye-Red and Mem Dye-Deep Red, PKH26 red fluorescent dye, PKH67 green fluorescent dye, 1,1′-dioctadecyl-3,3,3′,3′-tetramethylindo-carbocyanine perchlorate (DIL), and 2-(5-(1,3-dihydro-3,3-dimethyl-1-octadecyl-2H-indol-2-ylidene)-1,3-pentadienyl)-3,3-dimethyl-1-octadecyl-perchlorate (DID)) [31].

Exosome research has been significantly influenced by the work of Théry *et al*. [32], who laid the foundation for exosome isolation from cell culture supernatants and biological fluids. This offers a standardized approach for researchers, which has driven the refinement of isolation techniques to capture exosomes with specific functional attributes [32]. For example, while ultracentrifugation is considered a gold standard method, it may affect the integrity of exosomes due to the high-speed centrifugal forces.

The International Society for Extracellular Vesicles has contributed to the establishment of minimal experimental requirements for defining extracellular vesicles (EVs) and understanding their functions while also emphasizing the importance of standardized approaches for characterizing EVs [4, 33]. This aligns with recent publications where the challenges related to EVs isolation have been discussed along with emphasizing the need for standardization in the field [34]. Furthermore, it is essential to consider the biological properties and physiological functions of EVs to further appreciate the significance of isolation techniques [5]. These findings are consistent with a more comprehensive understanding of EVs including their function in cellular communication, physiological processes, and pathological states [34].

There are several methods for EVs/exosome-enriched fraction isolation and purification, including polymer precipitation, size-based isolation [size-exclusion chromatography (SEC) and ultrafiltration], ultracentrifugation, immunoaffinity capture, and microfluidics-derived methods [25, 32, 35, 36]. These methods have been adapted over time to accommodate the need for high-purity exosome samples. These methods have also been continually optimized [37] and the choice of isolation method may depend on available resources and downstream applications [33].

However, specialized or expensive equipment or reagents may not be available to some researchers, driving the choice of method which in turn can influence the purity and yield of exosomal isolations. While each of these methods yields exosome-enriched fractions, they can also be combined to increase the purity of the isolated exosomes or to address their intended use more specifically.

In this study, human embryonic kidney cells (HEK293) and human neuroblastoma cells (SH-SY5Y) were used to produce exosomes. HEK293 cells grow rapidly and are reported to produce a large number of exosomes [38, 39], while SH-SY5Y cells are slower growing and produce a lower number of exosomes. We have investigated two different culture media to stimulate cells to produce exosomes: Dulbecco's Modified Eagle Medium (DMEM)-based medium supplemented with exosome-depleted serum and DMEM-based medium without serum. In each of the methods, the fraction identified as containing exosomes is in fact “EVs or enriched with exosomes” as other particles inevitably co-isolate with them. The purpose of presenting these protocols is not to compare different methods but rather to provide a compendium of experimental methods so that researchers can choose the most appropriate method according to the available resources, facilities, and equipment in addition to the intended use of the exosomes. Researchers may find it necessary to adapt protocols or combine multiple methods to address resource limitations without compromising the quality of their exosome isolates [40]. In addressing this, we have brought these methods together into one source so that researchers have ready access to methodologies and troubleshooting information, along with steps to aid in the optimization of these methods for their specific situation.

## Materials and methods

### Cell culture and exosome enriched-containing medium collection

For culturing cells, 150-mm dishes or T125 flasks are recommended to ensure sufficient exosomes for isolation. In general, we used a plating density in 150-mm dishes which yielded confluency of 60–80% at 48-h post-plating (less for rapidly growing cells, more for slow-growing cells). Higher confluencies can result in cell death in which case there will be a loss of exosomes and the potential of isolating other membrane-bound structures or fragmented membrane vesicles in the extraction steps. As well, the use of serum-free medium may not be appropriate for the cell type being used and may also result in increased cell death. A shorter serum-free medium incubation may be appropriate for cells that suffer from cell death on serum starvation, in which case the exosome production period can be reduced to 24–36 h to prevent contamination of the medium with cellular fragments and contents resulting from cell death. The optimal confluency and thus the exosomal yield is also affected by the use of either serum-free or serum-containing media. If serum-free media is used, the suitable confluency may be about 70% for fast-growing cells and about 80% for slow-growing cells. Some cells may benefit from a gradual adaptation to serum-free medium by stepwise changing to 2–5% serum for 12–24 h and then moving to serum-free medium. When using medium containing exosome-depleted serum, it is better to adjust the confluency prior to this medium to approximately 60% and 70% for high- and low-doubling time cells, respectively. When seeding the cells, serum-containing medium can be used but care should be taken to wash the cells with PBS before adding serum-free or exosome-depleted serum media, so that all potential residual exosomes donated by the serum are removed prior to exosome production. Supplementary Fig. S1 shows the confluency and morphology of the HEK293 and SH-SY5Y cells when cells are at suitable confluency to be replaced with fresh media for exosome production, and again after further 48-h incubation when the cells are ready for conditioned media collection for the extraction of exosome.

**Figure 1.**
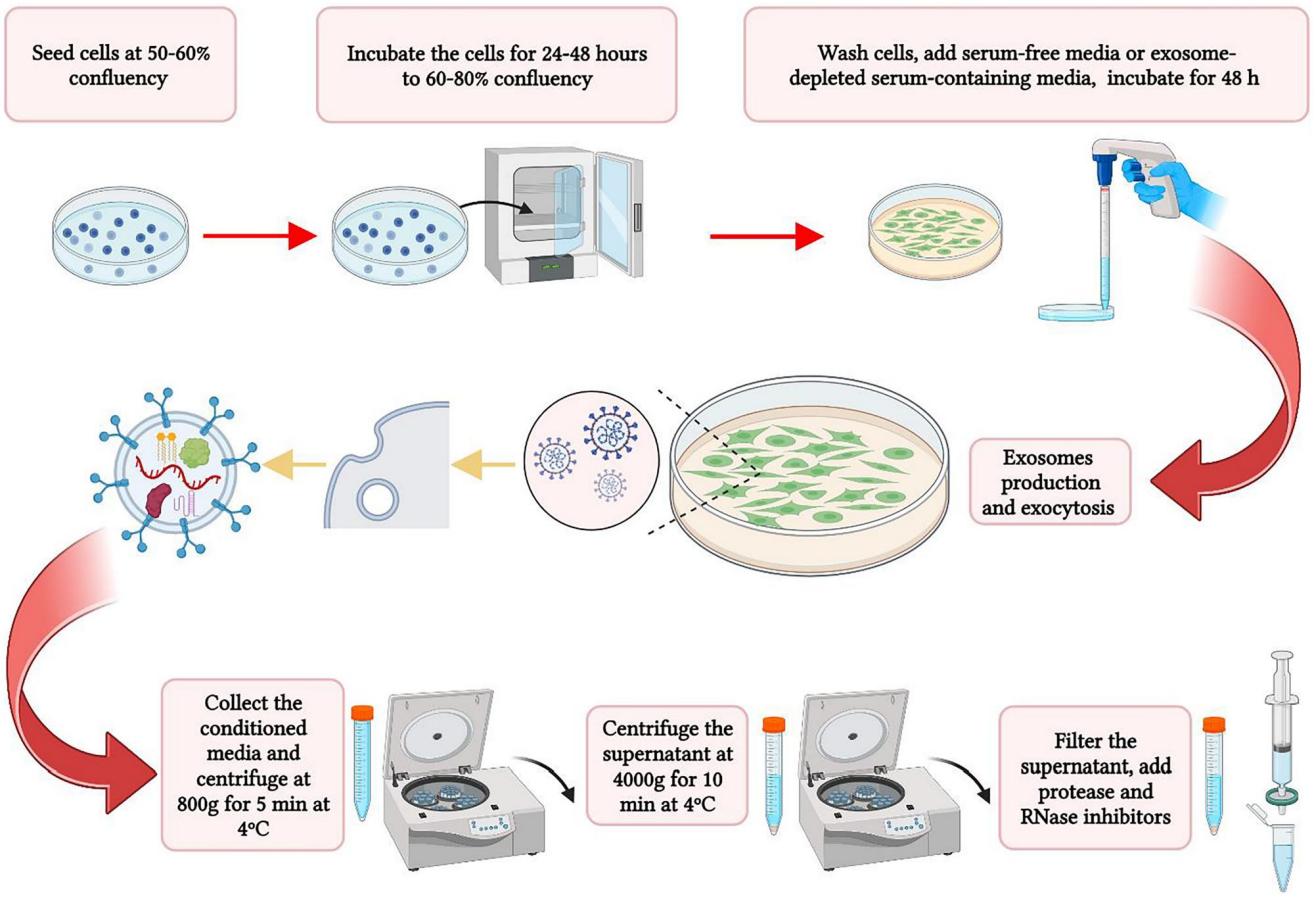
Conditioned media preparation. Schematic diagram for the preparation of exosome ECM. Figure created in BioRender.

All cells for this study were cultured on 150-mm dishes in DMEM (high glucose, with sodium pyruvate; Invitrogen) supplemented with 10% fetal bovine serum (FBS; Invitrogen) and penicillin/streptomycin (Invitrogen) and incubated at 37°C in 5% CO_2_ (Fig. 1). Two different culture media were used as conditioning media for expressing and isolating exosomes: (i) serum-free media (DMEM supplemented with penicillin/streptomycin without FBS) and (ii) exosome-depleted serum-containing media [DMEM supplemented with penicillin/streptomycin and serum that had been exosome depleted by centrifugation at 100 000*g* for 18 h followed by filtration through a 0.2-μm membrane low-protein binding filter (ThermoFisher)]. Cells were seeded at 3.5 × 10^4^ cell/cm^2^ (SH-SY5Y) or 2.6 × 10^4^ cell/cm^2^ (HEK293) in regular medium, incubated for 24 h and then washed three times with phosphate-buffered saline (PBS; Invitrogen). Media was then replaced with fresh serum-free media or media containing exosome-depleted serum (Fig. 1).

Exosomes can be isolated between 24 and 72 h following the replacement of the media for conditioning media as described above. While the yield of exosomes may be less after 24 h compared to 48 or 72 h, the latter time intervals may be associated with greater cell death and thus the optimum time for collection was defined by first developing a time course of the extent of cell death for each cell type and each culture condition. In this specific study, we collected the enriched-containing medium (ECM) after 48-h interval by removing the medium from the cultures using a sterile pipet followed by centrifugation at 800*g* for 5 min at 4°C to remove any cells and then centrifuging the supernatant at 4000*g* for 10 min at 4°C to remove residual debris. The supernatant was then retained and passed through a low protein binding 0.2-µm filter. For each 50 mL of filtered medium, 1 cOmplete protease inhibitor tablet (Roche) and 5 µl of RNAseOUT (Invitrogen) were added to prevent protein and RNA degradation. The resultant ECM can be used immediately or stored at −20°C until further processing to extract EVs.

### EVs isolation

#### Polyethylene glycol 8000 method

**Figure 2.**
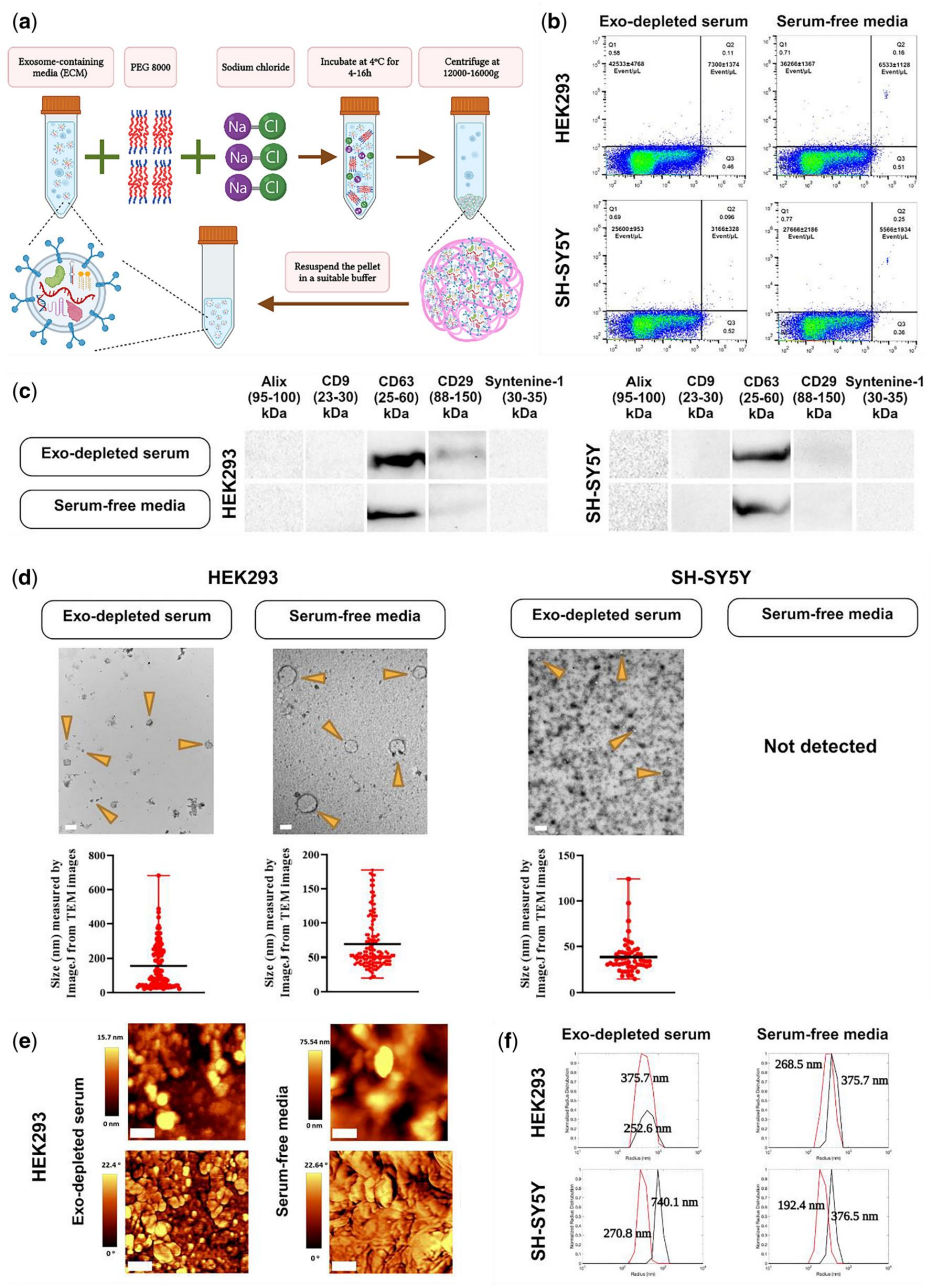
PEG 8000-based EVs extraction. a Schematic illustration. PEG 8000 results in increasing the sedimentation coefficient of EVs, thus allowing for isolation by centrifugation at a low speed. (b) Dot plot diagram-derived flow cytometry indicating the number of EVs probed with PE anti-CD9 antibody for either HEK293 or SH-SY5Y cells cultured in either DMEM containing exosome-depleted serum (Exo-depleted serum) or serum-free media [one representative plot is shown, *n* = 3, mean±sd; *y*-axis: PE fluorescence, *x*-axis: Long Angle Light Scatter (LALS)]. (c) Western blot analysis using five exosome-specific markers. CD63 and CD29 were positive for both cell lines. While CD63 was sharp and clear for both, CD29 was weak, especially for SH-SY5Y cells. (d) TEM images of EVs resulting from PEG 8000 method for both cell lines in exosome-depleted serum-containing media or serum-free media. Arrows indicate EVs enriched with exosomes. For each isolation condition, the particle size distribution was plotted using Image J software and the mean particle size illustrated. EVs from serum-free medium cultured SH-SY5Y cells were not detectable with TEM (scale bar: 1 µm for the first image, 100 nm for the others). (e) AFM images for HEK293-derived EVs with the top row showing the topography, while the bottom row is phase (scale bar: 400 nm). (f) Size measurement based on DLS for both cell types in both conditioned media [*y*-axis: normalized radius distribution; *x*-axis: radius (nm)]. The size was assessed at two time points, before sonication and after 3-min sonication on ice to disaggregate the accumulated EVs (black line: pre-sonication, red line: post-sonication) Figure 2a created in BioRender.

The ECM was supplemented with 50% polyethylene glycol (PEG) 8000 solution (in ddH_2_O) to a final concentration of 10% PEG, mixed well by inversion, supplemented with NaCl to a final concentration of 75 mM, and mixed again by inversion (Fig. 2a). This mixture was then incubated at 4°C for 4–16 h. Note that longer incubation times may increase exosome yield compared to very short incubation times. The sample was then centrifuged at 16 000*g* for 1 h at 4°C to precipitate the exosomal fraction. The pellet was then resuspended in 1× PBS. To remove any remaining contaminants in the pellet, this procedure was repeated once more. Final exosome-enriched fraction preparations were stored in 1× PBS, with short-term storage (days) at 4°C or longer-term storage (months) at −20°C or more preferably at −80°C [41], although in the latter scenario, caution should be used to employ low protein binding tubes.

#### Ultracentrifugation method

**Figure 3.**
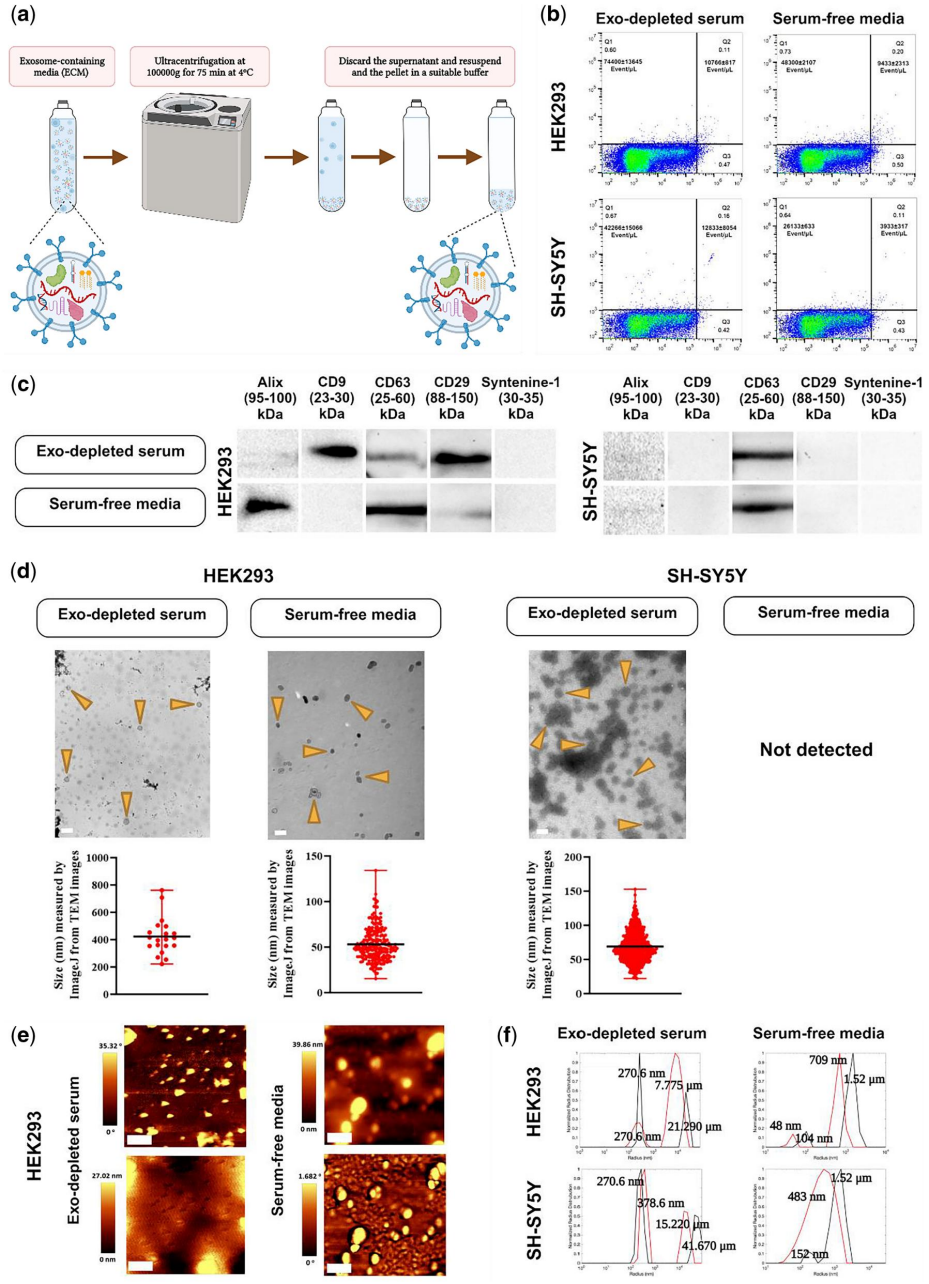
Ultracentrifuge-based EVs extraction. (a) EVs isolation on an ultracentrifuge basis is shown schematically. EVs are pelleted at 100,000*g* for 75 min at 4°C. (b) Dot plot diagram obtained from flow cytometry showing the quantity of EVs probed with PE anti-CD9 antibody for both cell lines cultivated in either DMEM containing exosome-depleted serum (Exo-depleted serum) or serum-free media (representative plot presented, *n* = 3, mean ± sd; *y*-axis: PE fluorescence, *x*-axis: LALS). (c) Western blot analysis employing five distinct exosome markers. Alix, CD9 (only found in exosome-depleted serum-containing media), CD63, and CD29 were observed in exosomal fractions derived from HEK293 cells. Additionally, Alix (very weak signal) and CD63 were detected in exosomal fractions obtained from SH-SY5Y cells. (d) TEM images of the ultracentrifugation-based EVs isolation for both HEK293 and SH-SY5Y cells in two culture media. Arrows indicate EVs enriched with exosomes. For each isolation condition, the particle size distribution was graphed using Image J software and the average particle size was shown. EVs from serum-free medium cultured SH-SY5Y cells were not detectable with TEM (scale bar: 1 µm for the first image, 100 nm for the others). (e) AFM images from the EVs isolated from HEK293 (topography: top images, and phase: bottom images, scale bar: 400 nm). (f) Normalized radius distribution of extracted EVs measured by DLS for HEK293 (top) and SH-SY5Y (bottom) cells in both culture media [*y*-axis: normalized radius distribution; *x*-axis: radius (nm)]. To disaggregate the collected EVs, the size was measured before and after 3 min of sonication (black line: pre-sonication, red line: post-sonication). Figure 3a created in BioRender.

The ECM was centrifuged at 100 000 g for 75–90 min at 4°C (Beckman Coulter^TM^, rotor type 70Ti) to pellet the EVs using heat-sealed tubes appropriate for the ultracentrifuge (Fig. 3a). Using a needle puncture technique, the supernatant was withdrawn from the tube leaving the exosome-enriched pellet in the bottom; the bottom of the tube was excised, and the pellet resuspended in 100 µL of PBS containing protease inhibitor and RNAseOUT on ice [working solution: 50 mL of filtered PBS, 1 cOmplete protease inhibitor tablet (Roche) and 5 µl of RNAseOUT (Invitrogen)]. Alternatively, the EVs may be resuspended in a suitable buffer using a clean needle/syringe and withdrawn from the tube without excising the bottom of the tube. The pellet containing the EVs was not allowed to dry to prevent damage to the EVs. To wash the EVs, the ultracentrifugation procedure was repeated. Increased solubility of the exosome-enriched pellet can be achieved by allowing the pellet to solubilize for 16–24 h at 4°C to ensure that all of the sample is resuspended. Note that the time and speed of ultracentrifugation may need to be altered following careful examination of the morphology of the EVs. We found that the EVs may be shrunken after spinning at 100 000*g* for 90 min; however, more spherical morphology was retained at 90 000*g* for 90 min or when spun at 100 000*g* for 75 min.

#### Ultrafiltration method

**Figure 4.**
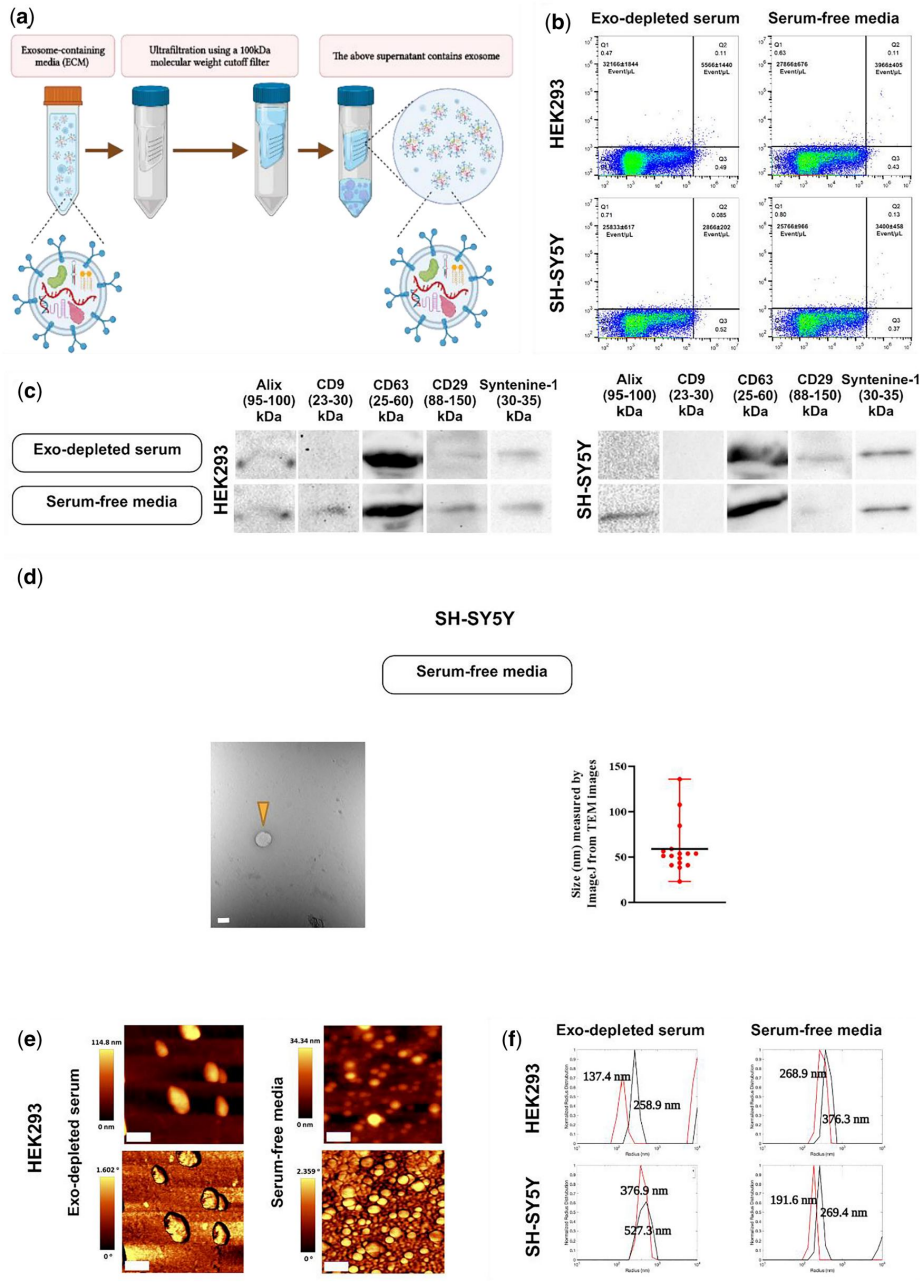
Ultrafiltration-based EVs extraction. (a) Schematic depiction. After the content of the secretome is passed through a 100-kDa filter membrane, the supernatant available on the upper side of the membrane contains exosomes-enriched fractions. (b) Flow cytometry employing dot plot diagram to detect the number of EVs probed with PE anti-CD9 antibody (one plot is shown as representative, *n* = 3, mean ± sd; *y*-axis: PE fluorescence, *x*-axis: LALS). (c) Western blot analysis to detect some exosomal markers. Alix, CD9 (except for exosome-depleted serum-containing media), CD63, CD29, and Syntenine-1 were positive for HEK293T cells, while Alix (except for exosome-depleted serum-containing media), CD63, CD29, and Syntenine-1 were positive for SH-SY5Y cells. (d) TEM analysis to observe the EVs morphology and integrity which was derived from ultrafiltration method for SH-SY5Y cell lines in serum-free media. Arrows indicate EVs enriched with exosomes. The particle size distribution was graphed using Image J software, and the average particle size was shown. EVs in both conditioned media for HEK293 cells and for exosome-depleted serum-containing media (Exo-depleted serum) for SH-SY5H cells were not detectable with TEM (scale bar: 100 nm). (e) Topography: top images, and phase: bottom images derived from AFM for the EVs isolated from HEK293 (scale bar: 400 nm). (f) Normalized radius distribution of DLS measurement for the EVs derived from HEK293 (top) and SH-SY5Y (bottom) cells in both culture media [*y*-axis: normalized radius distribution; *x*-axis: radius (nm)]. The size was assessed twice, before and after 3 min of sonication on ice (black line: pre-sonication, red line: post-sonication). Figure 4a created in BioRender.

**Figure 5.**
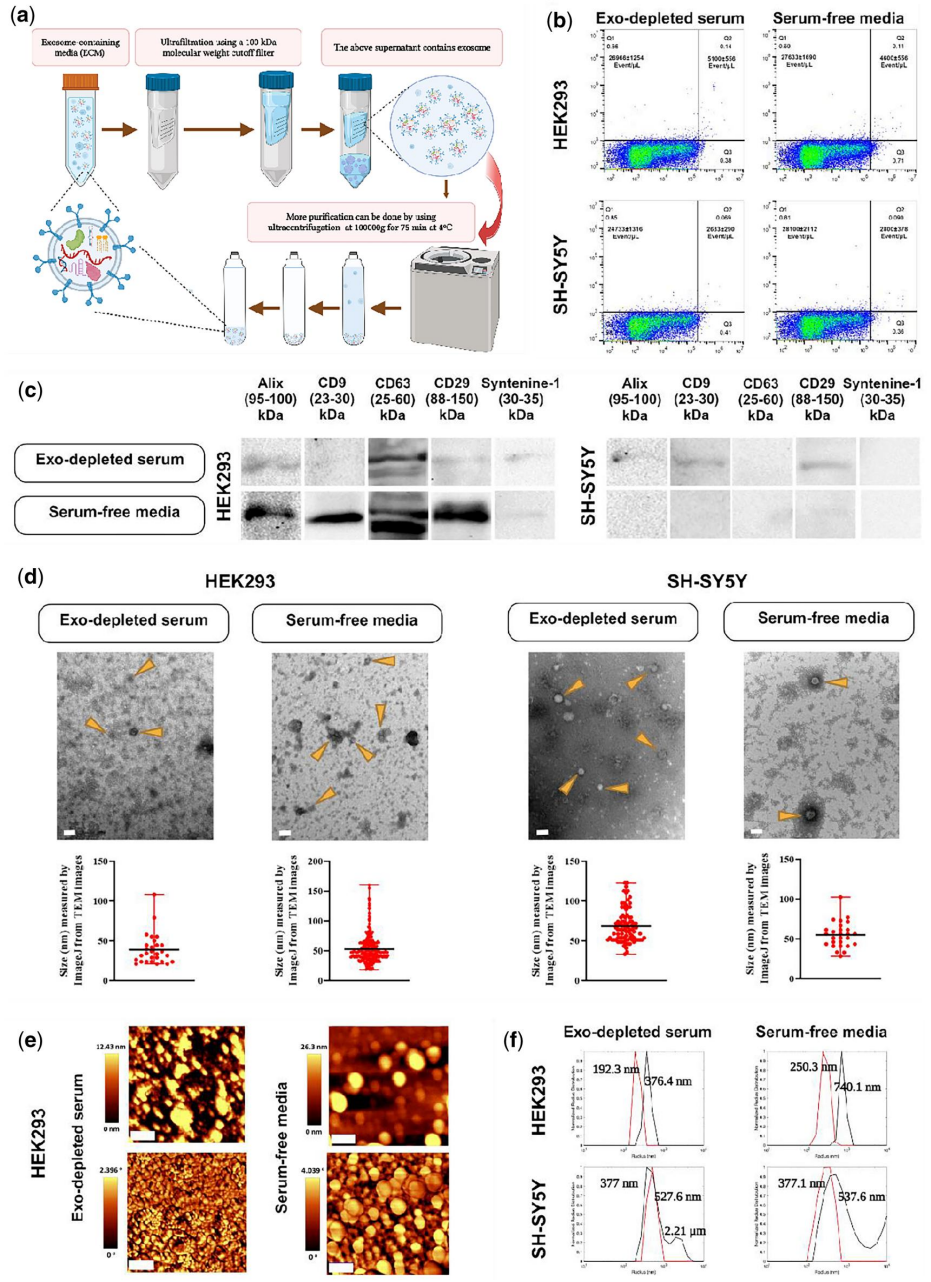
Ultrafiltration–ultracentrifugation-based EVs extraction. (a) Schematic illustration of ultrafiltration–ultracentrifugation-based EVs isolation. The content of the secretome was passed through a 100-kDa filter membrane followed by further purification of the supernatant derived from apical side of the membrane by centrifugation at 100,000*g* for 75 min at 4°C. (b) Dot plot diagram resulting from flow cytometry using PE anti-CD9 antibody to determine the number of EVs. The cells were cultured in DMEM containing either exosome-depleted serum (Exo-depleted serum) or serum-free media, and then employing flow cytometry the number of isolated EVs was calculated (one plot is displayed as an example, *n* = 3, mean ± sd; *y*-axis: PE fluorescence, *x*-axis: LALS). (c) Western blot analysis using exosomal marker antibodies. All exosome markers were detected for HEK293 cells at both conditioned media. For SH-SY5Y cells, however, Alix, CD9, and CD29 signals were detected in exosome-depleted serum-containing media, but very weak signals for CD63 and CD29 were detected in serum-free media. (d) TEM analysis to assess the morphology and integrity of EVs isolated from ultrafiltration–ultracentrifugation-based method for HEK293 and SH-SY5Y cells in two different culture media. Arrows indicate EVs enriched with exosomes. The particle size distribution was illustrated using Image J software for each isolation condition, and the average particle size was displayed (scale bar: 100 nm). (e) AFM analysis of EVs (topography: top images, and phase: bottom images) derived from HEK293 (scale bar: 400 nm). (f) EVs’ normalized radius distribution as determined by DLS for the EVs derived from HEK293 (top) and SH-SY5Y (bottom) cells in both culture media [*y*-axis: normalized radius distribution; *x*-axis: radius (nm)]. The size was measured twice, before and after 3 minutes of sonication on ice (black line: pre-sonication, red line: post-sonication). Figure 5a created in BioRender.

The ECM was subjected to ultrafiltration by passing through a 100-kDa molecular weight cutoff filter [Amicon^®^ Ultra-15 Centrifugal Filter Unit (UFC9100)]. Different filter units may have varying degrees of protein binding affinity and retention of EVs. Alternative filters with low protein binding affinity, such as the ThermoFisher Pierce^TM^ Protein Concentrator PES, 100K MWCO would be recommended. In this procedure, the material retained above the filter contains the exosome-enriched fraction (Fig. 4a). Depending on downstream techniques and required exosome purity, this fraction can be used as isolated or further purified (e.g. by ultracentrifugation, sucrose density gradient centrifugation or SEC) (Fig. 5a). Using this method, 15–20 ml of ECM could be concentrated to approximately 200–500 µl. In the event that further purification is desired, the materials derived from ultrafiltration can be subjected to ultracentrifugation. To do so, the 200–500 µl samples which were concentrated using ultrafiltration were further centrifuged at 100 000*g* for 75 min at 4°C. This step removes any extra materials included in the ECM including phenol-red, proteins, and so forth. This step can also be replaced with sucrose density gradient centrifugation [42] or SEC.

#### Commercial kit exosome purification

**Figure 6.**
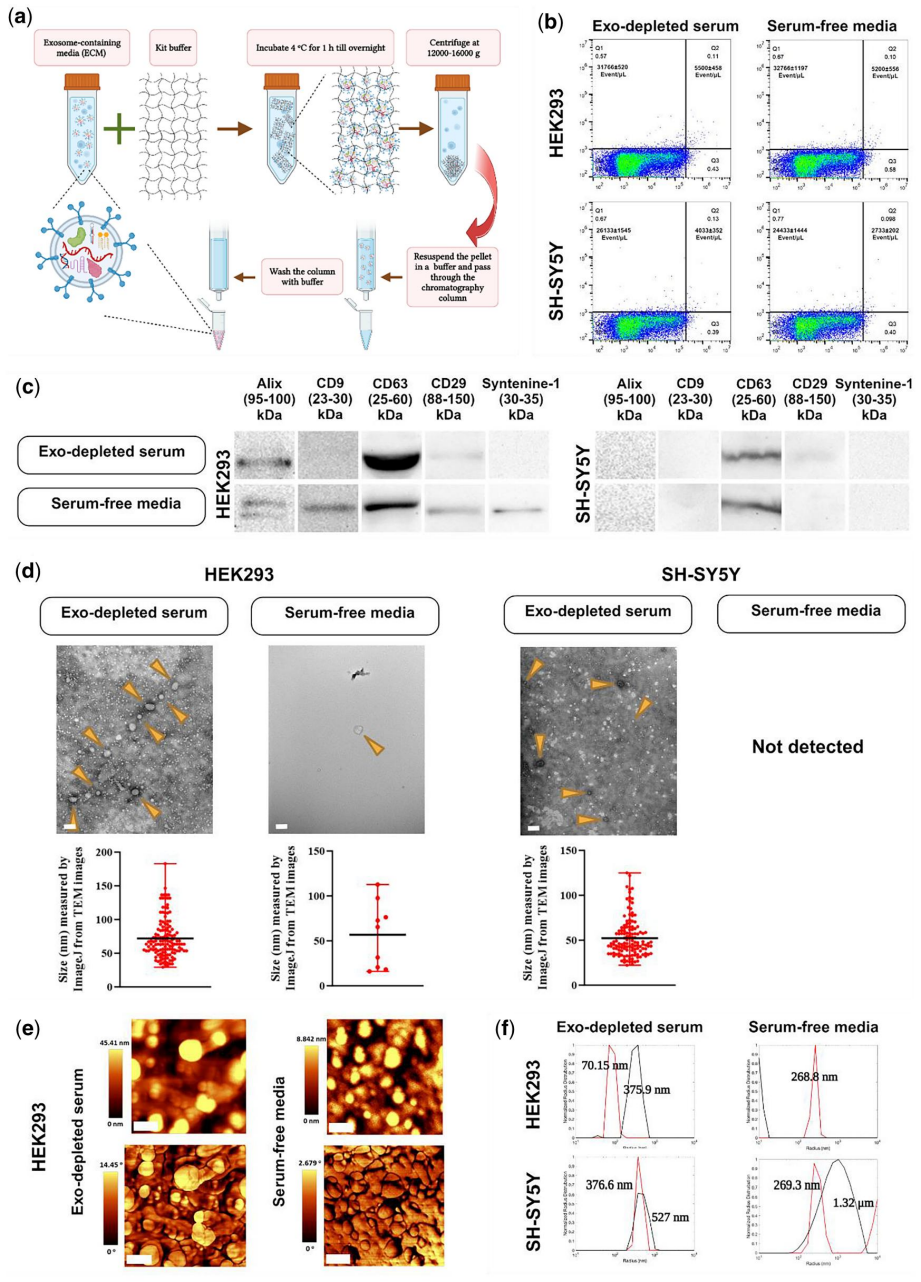
Kit-based exosome extraction. (a) Schematic diagram of the Exo-spin™ mini kit-based exosome isolation kit. Exosomes were dissolved in the kit buffer, precipitated by centrifugation, resuspended in PBS and then applied to SEC for more purification. (b) Flow cytometry diagrams (dot plot) counting exosomes. The exosomes derived from HEK293 and SH-SY5Y cells cultured in DMEM containing either exosome-depleted serum (Exo-depleted serum) or serum-free media were probed with PE anti-CD9 antibody for flow cytometry (one plot illustrative is shown, *n* = 3, mean ± sd; *y*-axis: PE fluorescence, *x*-axis: LALS). (c) Western blot analysis of exosome markers. All exosomal markers in the serum-free media, and Alix, CD63, and CD29 in exosome-depleted serum-containing media were positive for HEK293 cells, while only CD63 and CD29 (very weak signals) were positive for the SH-SY5Y cells. (d) TEM analysis to perceive the exosome morphology and integrity. Arrows indicate EVs enriched with exosomes. In each isolation condition, the particle size distribution was illustrated using Image J software and the mean particle size shown. Exosomes from serum-free medium cultured SH-SY5Y cells were not detectable with TEM (scale bar: 200 nm). (e) AFM images taken from HEK293-derived exosomes. The top row shows the topography, and the bottom row is phase (scale bar: 400 nm). (f) Normalized radius distribution of exosomes taken from DLS for HEK293 (top) and SH-SY5Y (bottom) cells cultured in both conditioned media (*y*-axis: normalized radius distribution; *x*-axis: radius (nm)). The size was assessed twice, before and after 3 min of sonication on ice (black line: pre-sonication, red line: post-sonication). Figure 6a created in BioRender.

There are several commercial kits for exosome isolation and purification from different biological samples or cell culture-conditioned media. These kits are usually based on either polymer precipitation in combination with SEC or filtration. While employing kits can provide pure exosomes quickly, the yield may be lower than that of “gold standard” methods such as ultracentrifugation and there is an associated cost which may be prohibitive for some labs. Here we employed a commercial kit [Exo-spin™ mini (Cell Guidance Systems LLC, USA)], that relies on both precipitation and SEC (Fig. 6a). Amongst the methods utilized in this article, this is the only one that employs SEC. Briefly, following the kit’s protocol, the ECM was mixed with the kit buffer, incubated at 4°C, and then centrifuged at 16 000*g* for 1 h, and finally purified by dissolving the pellet in 1× PBS and passing it through the column provided with the kit. The column was washed and the EVs recovered.

### Protein assay and Western blotting

Proteins contained within EVs or the lipid bilayer may not be detected by western blotting (WB) unless the EVs are permeabilized. A lysis buffer containing detergent such as NP40 or RIPA is required [25, 43–45]. Given this, EVs were resuspended and permeabilized in NP40 lysis buffer (50 mM Tris pH 8.0, 100 mM NaCl, 1 mM EDTA pH 8.0, 1% NP40/IGEPAL, 10% glycerol, supplemented with 1 protease inhibitor tablet (Roche)/50 ml volume). Alternatively, RIPA lysis buffer (50 mM Tris HCl, 150 mM NaCl, 1% NP40, 0.5% sodium deoxycholate, 0.1% SDS, 1 mM EDTA, and 0.01% sodium azide, pH 7.4 supplemented with 1 mM phenylmethylsulfonyl fluoride (PMSF)) can be used. The protein concentration was determined using the DC protein assay kit (Bio-Rad) according to the manufacturer’s instructions. Twenty microgram of total protein was then dissolved in Laemmli sample buffer, heated at 95°C for 5–10 min to denature the proteins, and then separated on a 12% sodium dodecyl–sulfate polyacrylamide gel electrophoresis (SDS–PAGE), followed by transfer to nitrocellulose membrane using transfer buffer (25 mM Tris, 192 mM glycine, 20% MeOH). The membranes were then washed in 1× Tris-buffered saline containing Tween-20 (TBS-T, 50 mM Tris, 150 mM NaCl, 1% Tween-20, pH 7.4), blocked in the same buffer supplemented with 5–10% skim milk (blocking buffer), and then incubated at room temperature (RT) with shaking for 1 h. Primary antibody (Table 1) in blocking buffer was then added and incubated overnight at 4°C. Membranes were washed in TBS-T three times, then probed with horseradish peroxidase (HRP)-conjugated secondary antibody (Table 2; 1:3000) in blocking buffer for 1 h at RT. The membrane was then washed in TBS-T three times and visualized using an enhanced chemiluminescence reagent (Perkin-Elmer) and a Bio-Rad Gel-Doc imaging system. When several protein epitopes needed to be evaluated consecutively, either multiple WB were prepared or membranes were stripped (20 mM glycine, 3 mM SDS, pH 2.2) for 15 min at 37°C followed by 20 min at RT, washed in TBS-T, and then reblocked for reprobing.

**Table 1.** Primary antibodies used herein for western blot and flowcytometry.

Antibodies	Dilution	Species	Used for	Company	Cat number
Alix	1:5000	Rabbit polyclonal IgG	WB	Proteintech	12422-1-AP
CD9	1:1000	Rabbit polyclonal IgG	WB	Stress Marq Biosciences	SPC-1272
CD63	1:1000	Rabbit polyclonal IgG	WB	Invitrogen	PA5-92370
CD29	1:5000	Rabbit polyclonal IgG	WB	GeneTex	GTX128839
Syntenine-1	1:1000	Mouse monoclonal IgG2a κ syntenin-1 antibody	WB	Santa Cruz	sc-515538
CD9	5 µg/ml	PE Mouse Anti-Human CD9 (M-L13) RUO	Flow cytometry	BD Biosciences	555372

**Table 2.** Secondary antibodies.

Antibodies	Dilution	Company	Cat number
Goat anti-Rabbit IgG (H+L)-HRP-conjugate	1:3000	Invitrogen	65-6120
Goat anti-Mouse IgG (H + L)-HRP-conjugate	1:3000	Bio-Rad	1706516

### Nanoscale flow cytometry

For flow cytometry, we used an R-Phycoerythrin (PE) anti-CD9 antibody to detect the number of EVs that express this marker on their surface. The EVs pellet derived from each of the protocols above was resuspended and diluted 1:100 in 1X PBS to a final volume of 1 ml. Samples were loaded on an Apogee A60 Microplus Nanoscale Flow Cytometer (Apogee Flow Systems Inc, UK) and data were analyzed using FlowJo Software. For samples from PEG isolation, ultracentrifugation, and kit-based methods, 10 µl of PBS-dissolved exosomal-containing pellets was incubated with 2 µl anti-CD9 antibody for 30 min in dark at RT, then diluted 1:100 in 1× PBS to a final volume of 1 ml, and subsequently analyzed using Flow cytometry. For the filtration method-derived sample, 10 µl of the materials derived from the top of the filter was incubated with 2 µl antibody for 30 min, diluted in 1× PBS to a final volume of 1 mL and then subjected to flow cytometry.

### Transmission electron microscopy analysis

For TEM, 5 µl of the exosome-enriched isolate was placed on a carbon-coated 400-mesh grid for 30 s. The grids were washed twice in water and then stained with 2% uranyl acetate for 30 s. Imaging was performed using a CM10 transmission electron microscope (Philips Electron Optics, Eindhoven, The Netherlands) at 80 kV and images were collected using a Hamamatsu Orca HRL Camera (Advanced Microscopy Techniques, Woburn, MA, USA). For TEM analysis of samples isolated using the PEG, ultracentrifugation, and kit-based methods where the EVs samples were in PBS, samples were diluted 1:1 in fixation buffer, and then subjected to TEM analysis. Samples from the filtration method (above the filter) were diluted 1:1 in fixation buffer for the TEM analysis. A fixation buffer (e.g. 2% glutaraldehyde and 2% paraformaldehyde in 0.1 M cacodylate buffer, pH 7.4) was used for TEM analysis.

### AFM

Twenty microliters of EVs sample was diluted to 60 µl in distilled water and drop cast onto borosilicate glass slides (Bio Nuclear Diagnostics, Inc, cat. No. LAB-033). Prior to drop-casting, the glass slides were ultrasonicated in isopropanol for 5 min, then in distilled water for 10 min, and then dried under nitrogen. The samples were dried for 2 h on the glass slides at RT until all water was evaporated, and a film of particles was left. These samples were placed on the scanning stage of a WiTec Alpha 300 AFM, set in tapping mode and equipped with PPP-NCHR-50 cantilevers (Nano-and-More, 125 ± 10 µm × 30 ± 7.5 µm × 4 ± 1 µm length × width × thickness) at 204–497 kHz resonant frequency and 10–15µm tip height, with force constants 10–130 N/m). Scans of 2 × 2µm^2^ were recorded over 256 × 256 pixels per image (∼8nm^2^/pixel).

### DLS

Twenty microliters of sample was diluted to 2000 µl in distilled water to meet the minimum volume requirement for the system. Samples were placed in a DLS apparatus (ALV GmbH) consisting of an integrated ALV/CGS-3 platform-based goniometer system, equipped with a He-Ne probe laser (22mW, 633 nm, Melles Griot) and an ALV-5000/EPP digital correlator attached to an ultrafast avalanche photodiode (APD). The autocorrelation function of scattered light intensity from the He-Ne laser was measured at 30^°^ from the incident light path for 10 s and averaged over three runs. Radii distributions were calculated using the ALV-Correlator Software (Ver. 3.0) built into the instrument, which assumes spherical particles. Instrument calibration was performed using an index-matching Vat (serial no. 324) and an additional Pt-100 temperature probe. As aggregation may have occurred during extraction and storage, they were sonicated in an ice bath for 3 min and then measurements were repeated.

### Experimental design and replication

Each isolation procedure was performed in three independent replicates. For each method, six 150-mm plates of cells were used with two plates allocated for TEM, two for WB, and the last two for flow cytometry, AFM and DLS. This also allowed us to assess the consistency and reproducibility of each method’s performance.

## Results

The procedures and results of exosome-enriched extraction from the five methods presented here are shown in Figs. 2–6. (Fig. 2: PEG-based, Fig. 3: ultracentrifugation-based, Fig. 4: ultrafiltration-based, Fig. 5: ultrafiltration-ultracentrifugation-based, Fig. 6: kit-based exosomes extraction). We used flow cytometry, WB, TEM, AFM, and DLS to analyze the isolated EVs. However, it is important to note that not all of these techniques may work to detect EVs. As we observed in our experiments, for some samples we did not detect EVs using TEM, likely due to the small numbers of EVs or dilution factors preventing detection. For WB, we employed five antibodies against exosome markers. However, not all epitopes were detected across each of the isolation techniques, suggesting that exosomal populations and protein markers likely differ and the user likely will need to optimize which antibodies to use depending on their source and population of exosomes. This may also be due to the low number of detectable exosomes for a particular detection method. Therefore, these identification techniques will need user optimization depending on the type of EVs being isolated, and the quantity, quality, and cellular source of the exosomes.

### PEG 8000 method

Using the PEG 8000 method (Fig. 2), the number of exosome-enriched isolates detected by flow cytometry for HEK293 cells was 42 533 ± 4768/µl for smaller particles and 7300 ± 1,347/µl for larger particles in depleted serum-containing media, and 36 266 ± 1367/µl for smaller particles and 6533 ± 1128/µl for larger particles in serum-free medium. However, exosome-depleted serum-containing media yielded 25 600 ± 953/µl and 3166 ± 328/µl for smaller and larger particles, respectively, and serum-free media yielded 27 666 ± 2186/µl for smaller and 5 566 ± 1934/µl for larger EVs enriched with exosomes with SH-SY5Y cells (Fig. 2b). CD63 and CD29 signals derived from WB were detectable in both cell types and medium conditions; however, CD29 signals were very weak, especially in the case of SH-SY5Y cells. Alix, CD9, and Syntenin-1 signals were not detectable (Fig. 2c). The EVs including exosomes were detected by TEM images for the HEK293 cells in both conditioned media and for SH-SY5Y cells for only exosome-depleted serum-containing media (Fig. 2d). For HEK293 cells in exosome-depleted serum-containing media, a variety of round structures with distinctive features were observed. These include structures with membrane extrusions, fragments, and small black dots, as well as staining clumps. For this cell line in serum-free media, a range of round structures with varying sizes and characteristics were observed. These structures include aggregated particles ranging from 10 to 100 nm, large round structures surrounded by a probable double membrane measuring 150–200 nm, and individual round particles with sizes around 80 nm, 50 nm, and smaller. These findings provide valuable insights into the characteristics, morphology, and variations of the observed round structures. For SH-SY5Y cells in exosome-depleted serum-containing media, a variety of round structures with diameters between 20 and 70 nm, and smaller 10 nm round dots were observed. Aggregates of round structures were also observed, with most of them appearing as tiny round spots. The size of vesicles obtained from TEM images which was calculated by ImageJ for this method for HEK293 cells regarding the exosome-depleted serum-containing media and the serum-free media were 156.12 ± 13.19 nm and 68.9 ± 3.26 nm, respectively. This value for SH-SY5Y cells was 38.63 ± 2.5 nm in an exosome-depleted serum-containing media (Fig. 2d).

We also determined the morphology using AFM (Fig. 2e) and the size using DLS (Fig. 2f) for both EVs isolated from the conditioned media. The images of AFM for HEK293 in exosome-depleted serum-containing media showed that there are some large aggregates in the sample, but most particles are of uniform size, well under 400 nm. In serum-free media, in addition to aggregated particles, there are also distinct particles that remain visible with a diameter of less than 400 nm. Further detail regarding the AFM images is provided in Supplementary Fig. S2a, b, and 2k, l including a line graph of particle distribution, mean particle size and phase difference plots. We assessed the size using DLS both pre- and post-sonication to determine if there was any clumping present. With HEK293-derived EVs in the exosome-depleted serum-containing media, an increased relative intensity of sample peak post-sonication indicates that a portion of the samples had aggregated, and a portion had not, and that sonication reduced the aggregation (Fig. 2f upper-left panel). Moreover, similar position and intensity of peaks indicate no significant aggregation of sample in the same cells in the serum-free media (Fig. 2f upper-right panel). For the SH-SY5Y cells-derived EVs in both culture media, there were some aggregations of a few particles before sonication (740.1 nm, 376.5 nm for exosome-depleted serum-containing media and serum-free media, respectively), and actual particle size was only slightly smaller (270.8 nm and 192.4 nm) (Fig. 2f lower-left and right panels).

### Ultracentrifugation method

The number of EVs detected by flow cytometry using the ultacentrifugation method (Fig. 3) were 74 400 ± 13 645/µl and 10 766 ± 817/µl for smaller and larger exosome-enriched particles in exosome-depleted serum-containing media and 48 300 ± 2107/µl and 9433 ± 2313/µl in serum-free media, respectively, for HEK293; these values were 42 266 ± 15 066/µl, 12 833 ± 8054/µl, and 26 133 ± 633/µl, 3933 ± 317/µl for SH-SY5Y cells (Fig. 3b). By WB analysis, Alix, CD9 (this marker was only detected in exosome-depleted serum-containing media), CD63 and CD29 signals were detected in HEK293 cells-derived exosomal fractions, and Alix and CD63 from SH-SY5Y-derived fractions (Fig. 3c). The EVs enriched with exosomes were detected in TEM images for this method in the HEK293 cells in both conditioned media and for SH-SY5Y cells only with exosome-depleted serum-containing media (Fig. 3d). For HEK293 cells in exosome-depleted serum-containing media, uniformly sized round particles were observed, as well as partially collapsed EVs. The presence of staining clumps in the background was evident. For the same cells in serum-free media, the images showed oval structures, surrounded by a double-layered membrane and well-separated without clumps. For SH-SY5Y in exosome-depleted serum-containing media, a range of fuzzy and grainy round structures of variable sizes was observed. The structures display a variety of appearances, including irregular shapes and aggregates, and a consistent substructure became apparent in some images. Additionally, well-defined smaller round structures around 50 nm were observed alongside larger structures approximately 100 nm in size. The size of vesicles obtained from TEM images which was calculated by ImageJ for this method for HEK293 for the exosome-depleted serum-containing media and the serum-free media were 422.88 ± 30.30 nm and 53.6 ± 1.23 nm, respectively. For the SH-SY5Y cells, this value was 68.84 ± 0.88 nm in an exosome-depleted serum-containing media (Fig. 3d).

We also assessed the morphology and size of EVs derived from HEK293 through AFM and DLS. Using AFM, we could detect EVs in samples from both conditioned media (Fig. 3e). Particles exhibited a distribution of diameters on the order of 200 nm from the HEK293 samples cultured in exosome-depleted serum-containing media. In serum-free media, particles aggregate somewhat into sizes larger than 400 nm. However, smaller particles are still present (Fig. 3e, see also Supplementary Fig. 2c, d, k, and l for particle distributions, mean particle size, and the phase difference plots). As with other methodologies, we assessed the size using DLS both pre- and post-sonication to determine if there was any clumping present (Fig. 3f). DLS results indicated the size of 270.6 nm for HEK293 sample cultured in exosome-depleted serum-containing media. This sample had little aggregation, and sonication had no effect. The larger peak is likely due to another component of the solution, which was reduced by sonication more easily showing a post-sonication smaller radius (Fig. 3f, upper left panel). For this cell line in the serum-free media, pre-sonication samples show a peak at 1.52 µm, suggesting that there are other components present in the samples. A 48–104 nm peak is the anticipated size, as corroborated with AFM (Fig. 3f, upper-right panel). The SH-SY5Y sample which was cultured in exosome-depleted serum-containing media showed an average particle radius slightly lower after sonication, indicating more aggregation in this sample. After sonication, the radius matches that of HEK293-derived EVs. Here again, we observed another component in the solution that is much larger and which breaks up upon sonication (Fig. 3f, lower-left panel). For the SH-SY5Y sample cultured in serum-free media, the peak at 1.52 µm is too large to be individual exosomes. After sonication, the peak at 483 nm is the sample size, and a 152-nm peak is the expected sample size we are looking for (Fig. 3f, lower right panel). It seems that the samples extracted by this method are either highly aggregated or have impurities other than exosomes.

### Ultrafiltration method

In the ultrafiltration-based EVs extraction method (Fig. 4), the number of EVs detected by flow cytometry for the HEK293 cells were 32 166 ± 1844/µl for smaller size particles, 5 566 ± 1440/µl for larger size particles in exosome-depleted serum-containing media, and 27 866 ± 676/µl and 3,966 ± 405/µl for smaller and larger size particles, respectively, in serum-free media. In SH-SY5Y cells, these values were 25 833 ± 617/µl, 2866 ± 202/µl, and 25 766 ± 966/µl, 3400 ± 458/µl (Fig. 4b). WB detected almost all exosomal markers used here except CD9 (for both culture media) and Alix (for exosome-depleted serum-containing media) in SH-SY5Y cells, as well as CD9 for exosome-depleted serum-containing media in HEK293 cell-derived fractions (Fig. 4c). Using TEM analysis, we could observe EVs in the SH-SY5Y sample culture in serum-free media with an average particle size of 58.97 ± 7.025 nm. In the images, round structures of varying sizes ranging between 30 and 100 nm were observed. TEM for other samples in this method were not detected (Fig. 4d) likely due to dilution factors resulting in too few EVs loaded onto the grid with volumes retained above the filter. The EVs in both culture media for HEK293 were easily detected by AFM. In exosome-depleted serum-containing media for the HEK293 cells, particles remain uniformly spaced, with a minimal level of aggregation, and uniform diameter. In serum-free media, however, some very large aggregates are on the order of micrometers. Nevertheless, a large number of particles remained isolated, with uniform size and diameter of closer to 100 nm (Fig. 4e, see also Supplementary Fig. 2 e, f, k, and l for particle distributions, mean particle size, and the phase difference plots). DLS analysis provided size (Fig. 4f) and demonstrated a peak of very large-sized contaminants, and our sample at 258.9 nm for HEK293 cells in exosome-depleted serum-containing media in both pre- and post-sonication samples. Post-sonication samples show there was some light aggregation that was broken up leading to a 137.4 nm peak (Fig. 4f, upper left panel). In the same cells cultivated in serum-free media, however, we detected some minor aggregation before sonication (376.3 nm), and the actual particle size was slightly smaller at 268.9 nm post-sonication (Fig. 4f, upper right panel). For the SH-SY5Y cells cultured in exosome-depleted serum-containing media, we observed a peak with lower intensity of pre-sonication which indicates that only a fraction of particles aggregated, while over half were not (Fig. 4f, lower-left panel), and in a serum-free media there was some aggregation of a few particles before sonication, and actual particle size was only slightly smaller (Fig. 4f, lower right panel).

### Ultrafiltration followed by ultracentrifugation method

Due to the fact that there appeared to be contaminants when using the ultrafiltration method, to provide further purification, we used an ultrafiltration–ultracentrifugation-based EVs extraction method (Fig. 5a). It is worth mentioning that this method can also be followed by other purification methods such as SEC or sucrose density gradient centrifugation. The number of EVs detected by flow cytometry for the HEK293 cells were 26 966 ± 1254/µl and 5100 ± 556/µl for smaller and larger size particles, respectively, in exosome-depleted serum-containing media, and 27 366 ± 1690/µl and 4600 ± 556/µl in serum-free media. For SH-SY5Y cells, these values were 24 733 ± 1316/µl, 2633 ± 290/µl and 28 100 ± 2112/µl, 2800 ± 378/µl, respectively (Fig. 5b). WB detected almost all markers used for HEK293 cells at both conditioned media. Alix, CD9, and CD29 signals were also detected for SH-SY5Y cells in exosome-depleted serum-containing media, but weak signals for CD63 and CD29 in serum-free media in this cell were seen (Fig. 5c). We could also detect EVs in all samples by TEM (Fig. 5d) with the average size of 38.97 ± 3.36 nm and 52.89 ± 1.32 nm from HEK293 cells in exosome-depleted serum-containing media and serum-free media, respectively. In the exosome-depleted serum-containing media, a few round particles ranging in size from 20–50 nm were identified with some appearing as small round structures and others displaying irregular shapes. In serum-free media, a variety of round structures of different sizes and characteristics were observed. These structures include small round structures ranging from 20–50 nm, with visible membranes and some stain aggregation. Some images also showed larger round structures up to 70 nm, along with aggregates containing round structures within them. Additionally, a mix of small and large round structures with fuzzy or extended appearances is present. Furthermore, single or small clumps of round structures, primarily measuring around 50 nm, were observed. Overall, these findings provide insights into the presence of round particles of varying sizes and shapes, along with the occurrence of background staining (Fig. 5d). Also from TEM images, SH-SY5Y cell-derived EVs sizes were detected to be 68.39 ± 2.13 nm, and 55.01 ± 3.09 nm from exosome-depleted serum-containing media and serum-free media, respectively. In both conditioned media, a range of round structures with varying sizes and appearances was observed. Some were covered with spikes or fuzzy structures (Fig. 5d).

AFM also confirmed the presence of EVs enriched with exosomes. For the HEK293 cells cultured in exosome-depleted serum-containing media, particles aggregated on a smaller scale, giving complexes on the order of 500 nm, with some particles remaining isolated. In the serum-free media, particles aggregated regularly on the order of 2000 nm; however, many particles remained isolated with uniform shape and small diameter (Fig. 5e; see also Supplementary Fig. 2 g, h, k, and l for particle distributions, mean particle size, and the phase difference plots). The size of particles was also calculated by DLS analysis (Fig. 5f). In HEK293 cells for both conditioned media, we perceived some minor aggregations before sonication (376.4 nm and 740.1 nm); however, the actual particles size were only slightly smaller (192.3 nm and 250.3 nm; Fig. 5f, upper left and right panels). EVs from SH-SY5Y cells cultured in exosome-depleted serum-containing media showed a second peak (2.21 µm) on the tail of the main pre-sonication peak (377 nm), showing there were some large aggregates which were broken up (Fig. 5f, lower left panel). In the same cells cultured in serum-free media, a wide pre-sonication peak (537.6 nm) indicates aggregates with a larger range of radii, and a second peak at much larger radius showing some particles formed large aggregates (Fig. 5f, lower right panel).

### Kit method

With the kit-based exosome extraction (Fig. 6a), flow cytometry indicated that the number of exosomes for the HEK293 cells were the same amount in both culture media (31 766 ± 520/µl, 32 766 ± 1197/µl for smaller size and 5500 ± 458/µl, 5200 ± 558/µl for larger size particles, respectively). These values for SH-SY5Y cells were 26 133 ± 1545/µl (smaller size particles) and 4033 ± 352/µl (larger size particles) in the exosome-depleted serum-containing media, and 24 433 ± 1444/µl and 2733 ± 202/µl in the serum-free media, respectively (Fig. 6b). WB analysis detected all protein markers for exosomes isolated from HEK293 cells in the serum-free media. For the exosome-depleted serum-containing media Alix, CD63, and CD29 signals were detected, while only CD63 and CD29 were detected for the SH-SY5Y-derived exosomes (Fig. 6c).

TEM analysis showed exosomes for HEK293 cells with an average particle diameter of 71.85 ± 2.5 nm and 56.81 ± 12.14 nm in the exosome-depleted serum-containing media and the serum-free media, respectively. Both isolates contained an assortment of circular formations displaying diverse dimensions and visual characteristics. For SH-SY5Y cells, we could detect exosomes in only exosome-depleted serum-containing media with round morphology with an average size of 52.26 ± 1.86 nm (Fig. 6d). AFM images showed EVs enriched with exosomes. In the exosome-depleted serum-containing media for the HEK293 cells, other than some large, irregular aggregates, particles stay densely packed but entirely isolated with uniform diameter of approximately 200 nm. In serum-free media, particles remained densely packed but isolated with a uniform diameter of roughly 200 nm; however, their shape is more ellipsoidal (Fig. 6e; see also supplementary Fig. S2 i–l for particle distributions, mean particle size and the phase difference plots). DLS analysis (Fig. 6f) determined the radii of exosomes from HEK293 cells cultured in the exosome-depleted serum-containing media with a pre-sonication peak at 375 nm, indicating that the sample was aggregated, while post-sonication radius is at 70.15 nm (Fig. 6f, upper left panel). In HEK293 cultured in serum-free media, the pre-sonication peak was below 10 nm, and after sonication a peak was seen at 268.8 nm. Taken together, it is very likely that there were large aggregates present (which would provide a peak off the right side of the graph) that were reduced during sonication (Fig. 6f, upper right panel). For the SH-SY5Y cells cultured in exosome-depleted serum-containing media, the lower intensity of pre-sonication peaks indicates that only a fraction of particles were aggregated, while over half were not. Sonication broke up all aggregates (Fig. 6f, lower left panel). For the same cells cultured in serum-free media, the pre-sonication peak is over 1 µM, and wide, indicating aggregates of a large range of radii were present (consistent with the results from the HEK293 cells in the same medium conditions). Sonication reduced the sample to a uniform peak at 269.3 nm (Fig. 6f, lower right panel).

## Discussion

The therapeutic and diagnostic applications of exosomes have increased exponentially in recent years [13, 46–48]. As we continue to understand their biogenesis and function, the ongoing development of reagents, techniques, and protocols to isolate and characterize exosomes has also become essential [33, 35, 49]. These advancements emphasize the necessity for well-established and standardized methods and have led to protocols for the enhanced isolation and characterization of exosomes [4, 33, 37]. The continuous optimization of these methods has led to the purification of exosomes from complex biological fluids, such as blood, urine, and tissue culture media, making them a valuable resource in translational research [50]. However, the choice for the suitable method in each laboratory should be made depending on the scientific topic and downstream applications as well as available resources/equipment, as there is not a single best isolation technique [33]. Here, we have considered the practical aspects and resource requirements of these methods to provide insights into how researchers can orient EVs isolation to their specific needs and available resources.

In this study, we have examined five methods for isolating EVs that vary in terms of complexity and equipment required. Other methodologies exist, including immunoaffinity capture approaches using antibodies which covalently bond to magnetic beads. This latter technique is appropriate for separating exosomes with a particular membrane protein marker expression, but is not convenient or cost-effective for numerous samples and can also capture off-target debris [25]. De Sousa *et al*. [34] emphasize that EVs isolation methods should be carefully chosen based on the available resources and the intended use of the EVs, which aligns with the consideration of convenience and cost–effectiveness mentioned here. For example, since immunoaffinity capture utilizes magnetic beads, it would potentially be problematic in a therapeutic setting in which magnetic resonance imaging was being used given that any residual ferrous material might contaminate the immunoaffinity-captured exosomes. To circumvent this problem, alternative affinity-captured techniques exist such as the use of lectins which will attach to specific saccharide residues (such as mannose) on the surface of exosomes. However, as many cells have saccharide residues on their surfaces, this method would likely produce a heterogeneous population of exosomes contaminated with other materials, and it is also still unknown whether this methodology captures all forms or a smaller sub-population of exosomes [51].

Microfluidics devices for exosome isolation have been developed which employ size-based or immune-affinity techniques, such as the use of anti-exosome-specific antibodies [52–54]. The processing complexity of these techniques is increased by the need for off-chip sample preparation processes such as reagent mixing or plasma extraction. The advantages of the microfluidics-derived chip isolation approach typically include swift sample processing, cheap cost, and portability [25]. However, low yield can be an issue for downstream applications. Hence, the microfluidic approach for exosome isolation is primarily used in diagnostics rather than clinical applications [36]. While this method is an example of advancing toward more efficient exosome isolation techniques, it may not be easily accessible to all researchers [34].

Of the methodologies employed in this paper, each has its own strengths and weaknesses. For instance, the precipitation method employs a carrier such as PEG 8000 to capture water molecules in the solution so that the less-soluble EVs are forced out of the solution and easily recovered by low-speed centrifugation. This is a convenient and swift procedure which can be accomplished with a standard microfuge centrifuge. However, co-precipitation of PEG or lipoproteins may occur, resulting in the extraction of exosomes with a low purity [25, 34, 51].

While the use of ultracentrifugation is also a well-accepted, easy method for isolating a significant number of relatively pure EVs, their structure may be altered due to shear forces [32]. Moreover, repetitive centrifugation may also damage the EVs and affect their quality [25]. Furthermore, this method requires access to an ultracentrifuge, which is a relatively expensive equipment that must be run for several hours, which may pose barriers for locales that may not have consistent access to stable electricity grids or funding levels required for purchasing expensive equipment.

Using ultrafiltration to extract and purify EVs is an extremely rapid method; however, if this method is used without the support of other methods for further purification (e.g. using ultracentrifugation, density gradient centrifugation or SEC), there will be impurities contaminating the sample. This method is more expensive than the PEG 8000 method.

Employing a commercial kit to isolate and purify exosomes is an easy and fast method using size exclusion columns that usually have a lower exosome extraction rate than PEG 8000 isolation or ultracentrifugation. However, the exosomes obtained are purer when combined-method kits (precipitation and chromatography) are used. This method is also considered an expensive method.

Examination of the EVs derived by any given isolation procedure is needed within any specific study’s purpose and depending on the cell type and conditioned medium being used. We found that TEM failed to detect EVs occasionally, and in particular from SY-SY5Y cells in serum-free media. These cells generally produce fewer exosomes than HEK293 cells. With ultrafiltration methods, there was a challenge to detect EVs because of sample dilution. These observations highlight the importance of optimizing isolation techniques under individual conditions. We also observed that not all markers may consistently be detected, suggesting that either different populations of exosomes are being detected, that there is masking of epitopes during the extraction techniques, or that the levels of markers are below our detection thresholds. Variations in antibody performance and blot stripping and reprobing may have contributed to less distinct WB results.

These observations emphasize the importance of considering the compromises between purity, yield, and cost when choosing an EVs isolation method. Each laboratory's specific resource constraints and research objectives should guide the selection of the most appropriate isolation technique [34]. This may involve combining methods, as our study suggests, to optimize EVs quality and quantity for different applications.

Each EVs isolation protocol presented here has its own resource requirements and possible adaptations for labs with limited resources. The PEG-based method, while cost-effective, can utilize lower-speed centrifuges and alternative PEG concentrations to alleviate resource constraints. The ultracentrifugation-based method, being equipment-intensive, may necessitate collaborations with other laboratories or alternative methods like PEG precipitation for resource-limited labs. Filtration methods are generally accessible (provided that there are no issues with ordering and procurement that may arise in less serviced areas of the world), but ensuring the proper filter cutoff size is crucial. Lower-cost filtration systems may be able to be used without compromising the overall effectiveness of the method. Commercial kits offer convenience but can be costly, resulting in cost becoming the prevailing consideration in choosing these methods in labs with smaller budgets. Researchers should assess equipment, budgets, and research needs to choose the most suitable method, potentially combining or modifying protocols to work within resource limitations, for example when pure exosomes are needed using kit-based protocols, or a combination of ultracentrifugation and SEC would be suitable because if ultracentrifugation is used alone, contamination of EVs isolates may be present (e.g. protein aggregates).

Storage and maintenance are critical issues for working with EVs. In our study, we used EVs or ECM immediately or stored them at 4°C for up to a week or at –80°C for prolonged duration. Previous studies have demonstrated that long-term storage at –80°C maintains small EVs stability, with minimal changes in size, concentration, and protein content although caution should be used to ensure that low protein binding tubes are utilized [41]. Short-term storage at 4°C may be suitable for some applications, but longer durations could lead to alterations in EVs characteristics. Moreover, freezing and thawing can impact the integrity and quantity of exosomes [41].

In conclusion, we have provided an analysis of five different techniques for exosomal analysis; however, there remains work to fully understand which of the EVs isolation and purification methods are most easily scaled to achieve large populations of exosomes.

## Supplementary Material

bpae009_Supplementary_Data

## Data Availability

The authors confirm that the data supporting the findings of this study are available within the article and/or its supplementary materials.
