# Supplementary material for: A methodological primer of extracellular vesicles isolation and characterization via different techniques
Source: Biol Methods Protoc. 2024 Feb 13;9(1):bpae009. doi: 10.1093/biomethods/bpae009 (PMC10902684; doi:10.1093/biomethods/bpae009)
Supplement: bpae009_Supplementary_Data [file bpae009_supplementary_data.pdf]

Supporting information

A methodological primer of extracellular vesicles isolation and characterization via different techniques

Farhang Aliakbari[1], Noah B. Stocek[2], Maxximuss Cole-André[2], Janice Gomes[1], Giovanni Fanchini[2,3], Stephen H. Pasternak[1,6], Gunna Christiansen[4], Dina Morshedi[5], Kathryn Volkening[1,6*], Michael J Strong[1,6*]

[1]Molecular Medicine Group, Robarts Research Institute, Schulich School of Medicine and Dentistry, University of Western Ontario, London, Ont, Canada; [2]Department of Physics and Astronomy, University of Western Ontario, London, Ont, Canada; and [3]Department of Chemistry, University of Western Ontario, London, Ont, Canada; [4]Department of Health Science and Technology, The Faculty of Medicine, Medical Microbiology and Immunology, Aalborg University, Aalborg, Denmark; [5]Bioprocess Engineering Department, Institute of Industrial and Environmental Biotechnology, National Institute of Genetic Engineering and Biotechnology, Tehran, Iran; [6]Department of Clinical Neurological Sciences, Schulich School of Medicine and Dentistry, University of Western Ontario, London, Ont, Canada.

***Correspondence**: Dr. Michael J. Strong (mstrong@uwo.ca) and Dr. Kathryn Volkening (kmcdouga@uwo.ca)

Supplementary Figure 1.

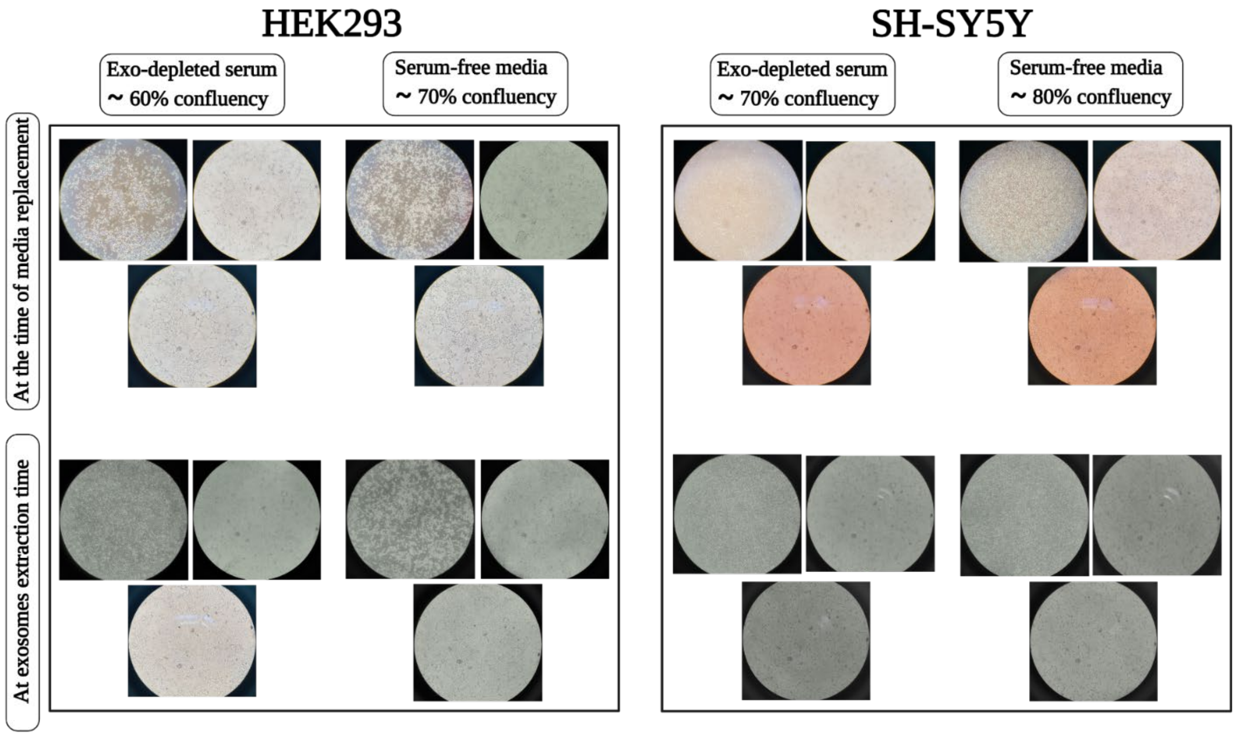

Supplementary Figure 1. The cells' confluency and morphology for EVs extraction. Cells morphology and confluency at the times of media replacement with fresh media which is specific for exosome release and EVs extraction using two culture media, DMEM high glucose supplemented with exosome-depleted serum and serum-free media. In each panel, the top left, right, and bottom images correspond to 4X, 10X, and 20X, respectively.

Supplementary Figure 2.

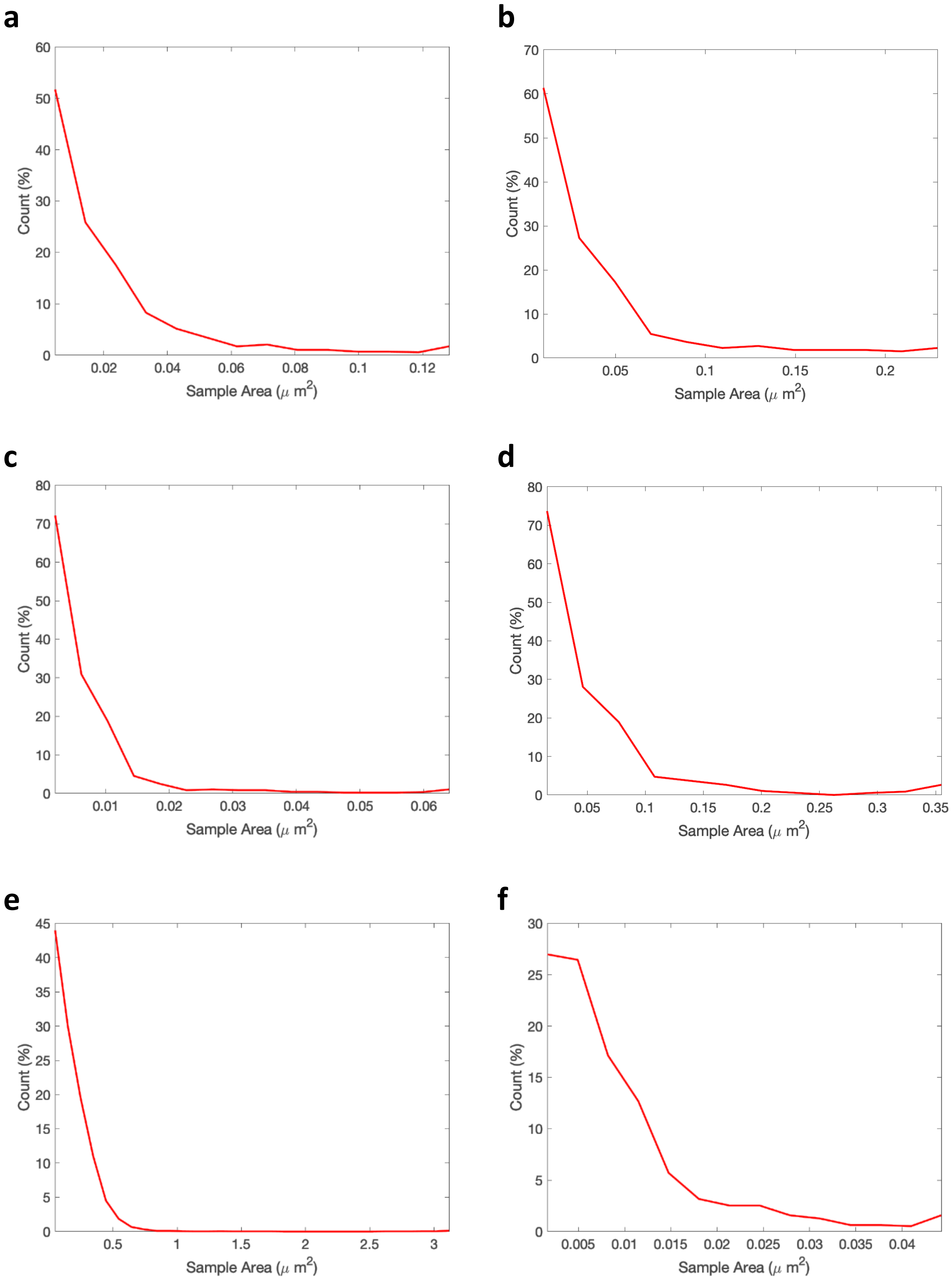

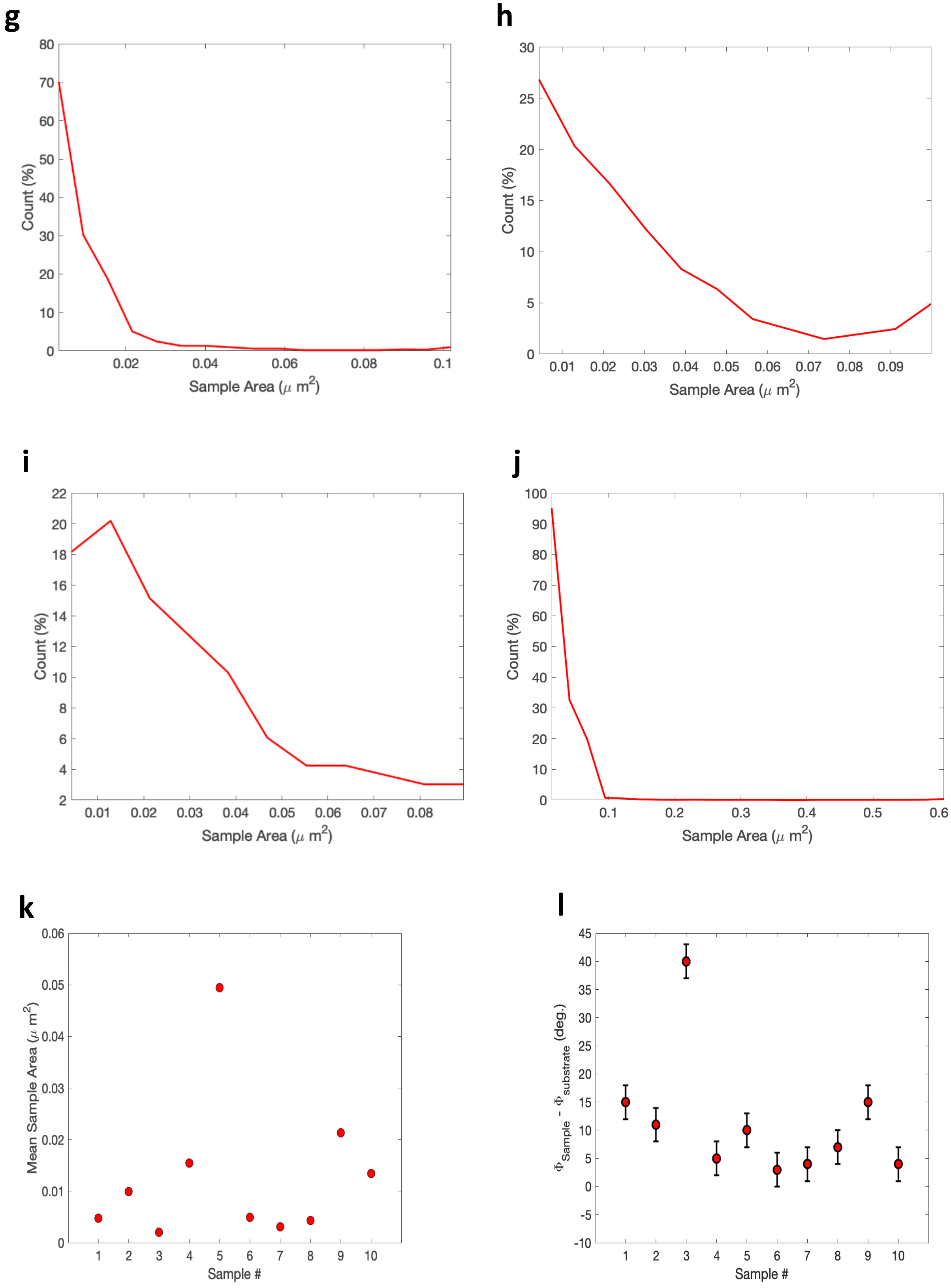

g
h
i
j
k
l
Count (%)
Sample Area (μ m2)
Mean Sample Area (μ m2)
Sample #
ΦSample - Φsubstrate (deg.)

Supplementary Figure 2. Line graph of distributions of the particles derived from AFM analysis for (a) PEG 8000-based method in exosome-depleted serum containing-media and (b) in serum-free media, (c) Ultracentrifuge-based method in exosome-depleted serum containing-media and (d) in serum-free media, (e) Ultrafiltration-based method in exosome-depleted serum containing-media and (f) in serum-free media, (g) Ultrafiltration-ultracentrifugation-based method in exosome-depleted serum containing-media and (f) in serum-free media, (i) Kit-based method in exosome-depleted serum containing-media and (j) in serum-free media. (k) The average particle area from each sample was calculated and the mean particle size for the samples is illustrated. (l) The phase difference plots between samples and glass. For sections k and l the number indicates the samples including 1 and 2: PEG 8000-based method in exosome-depleted serum containing-media and in serum-free media, respectively; 3 and 4: Ultracentrifugation-based method in exosome-depleted serum containing-media and in serum-free media, respectively; 5 and 6: Ultrafiltration-based method in exosome-depleted serum containing-media and in serum-free media, respectively; 7 and 8: Ultrafiltration-ultracentrifugation-based method in exosome-depleted serum containing-media and in serum-free media, respectively; 9 and 10: Kit-based method in exosome-depleted serum containing-media and in serum-free media, respectively.

Supplementary Table 1. Summary of EVs isolation methods for HEK293 cells

Method	Characterization Results	Cost	Time	Ease of Use
PEG 8000 method	- *Flow cytometry*: Exosome-depleted serum; small particles, 42,533 ± 4,768/µL and large particles, 7,300 ± 1,347/µL & serum-free media; small particles, 36,266 ± 1,367/µL and large particles, 6,533 ± 1,128/µL<br>- *WB*: Detected CD63 and CD29<br>- *TEM*: Vesicles with a variety of round structures with distinctive features<br>- *AFM*: Uniform-sized particles, some aggregation<br>- *DLS*: Some aggregation detected	Low	Fast	Easy
Ultracentrifugation method	- *Flow cytometry*: Exosome-depleted serum; small particles, 74,400 ±13,645/µL and large particles, 10,766 ± 817/µL & serum-free media; small particles, 48,300 ± 2,107/µL and large particles, 9,433 ± 2,313/µL<br>- *WB*: Detected Alix, CD9, CD63, and CD29<br>- *TEM*: Uniform-sized round and oval particles, staining clumps<br>- *AFM*: Uniform-sized particles, minor aggregation<br>- *DLS*: Large-sized contaminants detected before sonication	High	Moderate	Moderate
Ultrafiltration method	- *Flow cytometry*: Exosome-depleted serum; small particles, 32,166±1,844/µL and large particles, 5,566 ± 1,440/µL & serum-free media; small particles, 27,866 ± 676/µL and large particles, 3,966 ± 405/µL<br>- *WB*: Almost all markers detected except CD9 for exosome-depleted serum-containing media<br>- *TEM*: Not detected due to sample dilution<br>- *AFM*: Uniform-sized particles, some aggregation	Low-Moderate	Fast	Easy

	- *DLS*: Large-sized contaminants or aggregation detected before sonication			
Ultrafiltration-Ultracentrifugation method	- *Flow cytometry*: Exosome-depleted serum; small particles, 26,966 ± 1,254/µL and large particles, 5,100 ± 556/µL & serum-free media; small particles, 27,633 ± 1,690/µL and large particles, 4,600 ± 556/µL<br>- *WB*: Almost all markers detected<br>- *TEM*: Various round particles of different sizes along with some aggregates or clumps<br>- *AFM*: Particles with uniform shape, minor aggregation<br>- *DLS*: Minor aggregation detected before sonication	High	Moderate	Moderate
Kit method	- *Flow cytometry*: Exosome-depleted serum; small particles, 31,766 ± 520/µL and large particles, 55,500 ± 458/µL & serum-free media; small particles, 32,766 ± 1,197/µL and large particles, 5,200 ± 558/µL<br>- *WB*: Alix, CD63, and CD29 signals detected in exosome-depleted serum-containing media, while all markers detected in serum-free media<br>- *TEM*: Various circular structures with varying sizes and visual characteristics<br>- *AFM*: Densely packed, uniform particles, which become more ellipsoidal in serum-free media.<br>- *DLS*: large aggregates detected	Moderate-High	Fast	Easy

Supplementary Table 2. Summary of EVs isolation methods for SH-SY5Y cells

Method	Characterization Results	Cost	Time	Ease of Use
PEG 8000 method	- *Flow cytometry*: Exosome-depleted serum; small particles, 25,600 ± 953/µL and large particles, 3,166 ± 328/µL & serum-free media; small particles, 27,666 ± 2186/µL and large particles, 5,566 ± 1,934/µL<br>- *WB*: Detected CD63, CD29 (very weak)<br>- *TEM*: EVs detected for only exosome-depleted serum-containing media with round structures of variable sizes<br>- *DLS*: Some aggregations of a few particles detected	Low	Fast	Easy
Ultracentrifugation method	- *Flow cytometry*: Exosome-depleted serum; small particles, 42,266 ± 15,066/µL and large particles, 12,833 ± 8,054/µL & serum-free media; small particles, 26,133 ± 633/µL and large particles, 3,933 ± 317/µL<br>- *WB*: Detected Alix, CD63<br>- *TEM*: Round structures of variable sizes in exosome-depleted serum-containing media<br>- *DLS*: Very large-sized contaminants or highly aggregated detected before sonication	High	Moderate	Moderate
Ultrafiltration method	- *Flow cytometry*: Exosome-depleted serum; small particles, 25,833 ± 617/µL and large particles, 2,866 ± 202/µL & serum-free media; small particles, 25,766 ± 966/µL and large particles, 3,400 ± 458/µL<br>- *WB*: Almost all markers detected except CD9 (for both culture media) and Alix (for exosome-depleted serum-containing media)<br>- *TEM*: Round structures of varying sizes for serum-free media, and not detected for exosome-depleted	Low-Moderate	Fast	Easy

	serum-containing media due to sample dilution<br>- *DLS*: Aggregation was observed in only a fraction of particles			
Ultrafiltration-Ultracentrifugation method	- *Flow cytometry*: Exosome-depleted serum; small particles, 24,733 ± 1,316/μL and large particles, 2,633 ± 290/μL & serum-free media; small particles, 2,8100 ± 2,112/μL and large particles, 2,800 ± 378/μL<br>- *WB*: Alix, CD9 and CD29 in exosome-depleted serum-containing media detected, weak signals for CD63 and CD29 in serum-free media detected<br>- *TEM*: Various round particles of different sizes and appearances, some were covered with spikes or fuzzy structures<br>- *DLS*: Some large aggregates detected before sonication	High	Moderate	Moderate
Kit method	- *Flow cytometry*: Exosome-depleted serum; small particles, 2,6133 ± 1,545/μL and large particles, 4,033 ± 352/μL & serum-free media; small particles, 24,433 ± 1,444/μL and large particles, 2,733 ± 202/μL<br>- *WB*: Detected CD63 and CD29<br>- *TEM*: Various round particles of different sizes detected only in exosome-depleted serum-containing media<br>- *DLS*: Only some particles formed aggregates in exosome-depleted serum-containing media, while in serum-free media, a wide range of aggregate sizes was observed.	Moderate-High	Fast	Easy
